# 基于智能化分时区间采集技术的液相色谱-串联质谱法测定全血中磷脂酰乙醇及人群水平调查

**DOI:** 10.3724/SP.J.1123.2022.06025

**Published:** 2023-02-08

**Authors:** Zhaoyang LIU, Jun DONG, Hongxia LI, Ruiyue YANG, Zhiyu SHAO, Siming WANG

**Affiliations:** 北京医院，国家老年医学中心，国家卫生健康委北京老年医学研究所，国家卫生健康委老年医学重点实验室，中国医学科学院老年医学研究院，北京 100730; Beijing Hospital，National Center of Gerontology，Beijing Geriatrics Institute of National Health Commission，Key Laboratory of Geriatrics of National Health Commission，Institute of Geriatric Medicine，Chinese Academy of Medical Sciences

**Keywords:** 高效液相色谱-串联质谱, 智能化分时区间采集模式, 磷脂酰乙醇, 酒精摄入监测, high performance liquid chromatography-tandem mass spectrometry （HPLC-MS/MS）, intelligent scheduled time-zone acquisition technology, phosphatidylethanol （PEth）, alcohol intake monitoring

## Abstract

磷脂酰乙醇是具有潜在临床应用价值的酒精摄入生物学标志物，准确测定其含量可以为酒精摄入监测提供客观、量化的重要参考依据。本研究建立了一种基于智能化分时区间采集技术的液相色谱-串联质谱分析方法，能够同时实现对人全血样品中18种磷脂酰乙醇的准确测定。采用甲醇-甲基叔丁基醚-水作为萃取体系，选用XBridge C18色谱柱，以2.5 mmol/L乙酸铵异丙醇溶液和2.5 mmol/L乙酸铵水溶液-乙腈（50∶50， v/v）为流动相进行梯度洗脱，采用电喷雾离子源、智能化分时间段-负离子多反应选择离子监测模式。经验证，该方法线性关系良好，相关系数≥0.9998，线性范围在10~2500 ng/mL之间，检出限和定量限分别为0.7~2.8 ng/mL、2.2~9.4 ng/mL，加标回收率在91.0%~102.2%之间，日内精密度和日间精密度在0.4%~7.4%之间。该方法简便、快速、精密，智能化分时区间采集方法为每个离子通道分配合适的扫描时间段，增加了每个目标物离子通道的有效采集时长，提高了各目标分析物的信号响应，从而实现了对人全血样本中18种磷脂酰乙醇的有效分析测定。使用本方法测定了359名有规律饮酒习惯的志愿者全血样本，样本中的总磷脂酰乙醇含量范围为51.13 ng/mL~2.89 μg/mL，平均为363.16 ng/mL。磷脂酰乙醇16∶0/18∶1、16∶0/18∶2是两个丰度最大的同系物，平均含量分别为74.21和48.75 ng/mL，各约占总量的20.43%和13.42%。Spearman相关性分析结果显示，磷脂酰乙醇之间相关性良好，并与临床现有酒精生物学标志物*γ*-谷氨酰转肽酶呈正相关；同时磷脂酰乙醇与肝肾功能相关临床生化指标显著相关。所建方法可以准确、精密检测人血磷脂酰乙醇含量，能够客观、可靠、量化反映人体酒精摄入状况，并可为临床酒精摄入监测提供有潜在应用价值的分析手段。

## Abstract (en)

Alcohol intake is an important risk factor for cardiovascular disease， liver disease， and diabetes. The accurate and objective evaluation of alcohol intake is important for disease prevention and intervention， as well as alcohol intake monitoring. Phosphatidylethanol （PEth） is a potential clinical biomarker of alcohol consumption. Monitoring PEth levels can provide an objective and quantitative basis for alcohol intake studies. Unlike other current alcohol biomarkers， PEth can only be produced in the presence of alcohol. Therefore， PEth is highly specific for alcohol intake and not affected by confounding factors， such as age， gender， hypertension， kidney disease， liver disease， and other comorbidities. Because of its long half-life and high specificity for alcohol intake， PEth may be used as a tool for monitoring drinking behavior in the clinical， transportation， and other fields. Given rapid developments in mass spectrometry technology over the past decade， liquid chromatography-tandem mass spectrometry （LC-MS/MS） has become the preferred method for PEth detection. However， most current LC-MS/MS methods focus on the determination of one or several PEth homologs， and the number of PEth homologs that can be determined simultaneously is relatively limited. Moreover， the detection capacity of the available methods remains insufficient， and their analytical sensitivity for some PEth homologs must be further improved. In this study， a novel LC-MS/MS method based on an intelligent scheduled time-zone negative multiple reaction monitoring acquisition technology （Scheduled-MRM） was developed. The technology monitors two ion channels in each PEth to ensure reliable results and can quantify 18 PEth homologs in human whole blood simultaneously. Methanol-methyl *tert*-butyl ether-water was used as the extraction system. An XBridge C18 column （100 mm×2.1 mm， 3.5 μm） was selected for gradient elution with 2.5 mmol/L ammonium acetate isopropanol solution and 2.5 mmol/L ammonium acetate aqueous solution-acetonitrile （50∶50， v/v） as the mobile phases. Negative electronic spray ionization in scheduled-MRM mode was applied for MS/MS detection. The method was validated to have a linear range of 10-2500 ng/mL with correlation coefficients greater than 0.9999. The limits of detection and quantification were 0.7-2.8 and 2.2-9.4 ng/mL， respectively， and the spiked recoveries ranged from 91.0% to 102.2%. The method was confirmed to be simple， rapid， and precise， and subsequently validated for the measurement of 18 PEth homologs in human blood. Scheduled-MRM can assign a suitable scan time to each ion channel and effectively reduce the number of concurrent ion pairs monitored per unit time. This technology overcomes the problem of insufficient dwell time caused by an excessive number of ion channels， thereby avoiding the redundant monitoring of non-retention times. Scheduled-MRM significantly improved the detection sensitivity， data acquisition quality， and signal response of the proposed method. Whole blood samples from 359 volunteers with regular drinking habits were analyzed using this method. The total PEth concentrations ranged from 51.13 ng/mL to 2.89 μg/mL， with a mean of 363.16 ng/mL. PEth 16∶0/18∶1 and 16∶0/18∶2 were the two most abundant homologs， with mean concentrations of 74.21 and 48.75 ng/mL， accounting for approximately 20.43% and 13.42%， respectively， of the total PEth. Spearman correlation analyses showed that the PEth homologs correlated well with each other， *γ*-glutamyltransferase， a clinically available biological marker of alcohol， and other clinical biochemical parameters related to liver and kidney function. Overall， the method was demonstrated to be sensitive， precise， and accurate； thus， it may be an effective tool for monitoring alcohol intake in the clinical and other fields.

酒精摄入是全球范围内、跨越不同种群普遍存在的生活方式之一^[1]^，大规模的流行病学前瞻性研究认为，酒精摄入是心血管疾病、肝脏疾病、糖尿病的重要危险因素^[2,3]^。准确客观评价乙醇摄入量是疾病防治与干预、酒精摄入监测的重要依据。由于临床自述饮酒量的诊断效率有限且极易产生偏倚，客观性不足，而目前常规临床检验与酒精摄入相关的指标如*γ*-谷氨酰转肽酶、糖缺失性转铁蛋白等可能会由于其他内外因素和疾病状态而异常^[4,5]^。近年来，有部分研究开始尝试使用生物学标志物来评价乙醇摄入水平。

磷脂酰乙醇（phosphatidylethanol， PEth）是磷脂酰胆碱在乙醇存在条件下经磷脂酶D酶解的特殊代谢产物，其半衰期约为5天^[5⇓-7]^。与目前其他酒精生物标志物相比，PEth只在乙醇存在下才能生成，因此其对乙醇摄入具有极高的特异性，不受年龄、性别、体内其他代谢物或其他合并症（如高血压、肾病或肝病）等混杂因素的影响^[5]^。由于PEth拥有较长的半衰期，且对乙醇摄入具有极高的特异性，因此PEth作为酒精摄入标志物已引起人们的广泛关注。PEth由甘油磷脂骨架结构和两条脂肪酸长链组成，其脂肪酸链通常为含有14~22个碳原子以及0~6个数量不等的双键^[8,9]^，侧链的不同使PEth有许多分子种类^[8]^。

建立以PEth类物质为生物学标志物的酒精摄入准确评价方法有助于为饮酒相关疾病的临床研究和防治提供可靠数据，为乙醇滥用监测与干预提供保障，具有重要的社会、医疗和科学意义。PEth的检测技术经历了薄层色谱法^[10]^、气相色谱-串联质谱法^[11]^、高效液相色谱法^[12]^、液相色谱-串联质谱法（LC-MS/MS）^[13,14]^等多个阶段。近十年来随着质谱技术的快速发展，LC-MS/MS已成为目前检测PEth的首选方法，其可以根据脂肪酸侧链长度及双键数区分不同种类PEth并进行鉴定和定量^[4,15⇓-17]^。Aboutara等^[13]^在近期研究中建立了利用LC-MS/MS分析6种PEth同系物的方法，在对该方法进行全面验证后，对57例饮酒志愿者的干血斑样本进行测定，结果显示该方法分离良好、检测灵敏、定量可靠。Schröck等^[18]^通过在线SPE-LC-MS/MS对血液中的PEth16∶0/18∶1和16∶0/18∶2进行分析，用以评估摄入一定量酒精后PEth的代谢半衰期，结果显示PEth可以在饮酒后长达12天内被检测到，证明PEth在酒精摄入监测中具有潜在的应用价值。虽然LC-MS/MS应用于PEth检测的报道在逐渐增多，但目前大多数的LC-MS/MS方法都集中在测定一个或若干个PEth同系物上，能够同时测定的PEth同系物种类较少，检测容量仍显不足，且部分PEth同系物检测灵敏度也有待提高。针对上述问题，本研究采用电喷雾离子源、智能化分时区间-负离子多反应选择离子监测（Scheduled-MRM）技术，建立了一种简单、精密的液相色谱-串联质谱分析方法，同时测定全血样本中18种PEth同系物。智能化分时区间采集技术可以为每个离子通道分配合适的扫描时间段，增加每个目标物离子通道的有效采集时长，提高各目标物的响应和方法灵敏度。我们应用所建方法研究了临床饮酒志愿者全血中PEth含量及分布情况，探索其与肝肾功能相关临床生化指标的关系，为临床酒精摄入相关疾病研究提供客观的参考依据。

## 1 实验部分

### 1.1 仪器、试剂与材料

#### 1.1.1 仪器与试剂

LC-MS/MS包括Agilent 1260高效液相色谱仪（美国Agilent公司）、SCIEX QTRAP 5500质谱仪和Analyst 1.6.3数据处理软件（美国SCIEX公司）。MicroLab 500型稀释器（美国Hamilton公司），液体滚动混合器与Reacti-Vap蒸发器（美国Thermo Fisher Scientific公司）， WS100-D振荡器（德国Wiggens公司）， 3-18K离心机（德国Sigma公司）。

标准品：PEth16∶0/18∶1 （美国Enzo Biochem公司），PEth18∶0/18∶1、PEth18∶0/18∶2、PEth18∶1/18∶1、PEth16∶0/20∶4 （美国Echelon Biosciences公司），PEth16∶0/18∶2、PEth16∶0/16∶0 （美国Avanti Polar Lipid公司）。内标物：d5-PEth16∶0/18∶1、d5-PEth16∶0/18∶2、d5-PEth18∶0/18∶1、d5-PEth18∶0/18∶2、d5-PEth16∶0/20∶4 （美国Echelon Biosciences公司），磷脂酰丙醇16∶0/16∶0 （phosphatidylpropanol 16∶0/16∶0）、磷脂酰丁醇18∶1/18∶1 （phosphatidylbutanol 18∶1/18∶1）（美国Avanti Polar Lipid公司）。

有机溶剂包括甲醇、异丙醇、乙腈和甲基叔丁基醚（美国Thermo Fisher Scientific公司）。乙酸铵购自美国Sigma-Aldrich公司。水为本实验室自制超纯水。

#### 1.1.2 全血样本

征集359名有规律饮酒习惯的志愿者，采集静脉血于抗凝采血管中，分装后于-80 ℃保存。临床肝肾功能常规检验指标由临床检验数据系统直接调取。本研究获得北京医院医学伦理委员会的批准，志愿者均签署了知情同意书。

### 1.2 实验方法

#### 1.2.1 标准溶液和内标溶液的配制

以异丙醇为溶剂，精密配制质量浓度为10、50、100、250、500、1000、2500 ng/mL的7种PEth系列混合标准溶液，吸取1 mL分装于安瓿瓶中，于-20 ℃保存。7种内标用异丙醇配制成终质量浓度为250 ng/mL的内标混合溶液，吸取1 mL分装于安瓿瓶中，于-20 ℃保存。

#### 1.2.2 样本制备

将全血样品、标准溶液和内标溶液解冻、均质化并平衡至室温。使用稀释器精密吸取25 μL标准溶液或全血样品到安瓿瓶中，并加入25 μL内标混合溶液，加入600 μL甲醇沉淀蛋白质后，加入1200 μL甲基叔丁基醚，随后向混合物中加入600 μL水。将混合物振荡10 min，于4000*g*、4 ℃离心10 min。移取800 μL上清液，氮气吹干后用300 μL流动相进行复溶，随后采用LC-MS/MS进行分析。

#### 1.2.3 色谱条件

采用Waters X Bridge C18色谱柱（100 mm×2.1 mm， 3.5 μm）。流动相为A （2.5 mmol/L乙酸铵异丙醇溶液）和B （2.5 mmol/L乙酸铵水溶液-乙腈（50∶50， v/v）），以300 μL/min的流速进行梯度洗脱分离，梯度如下：0~3 min， 66%A； 3~3.4 min， 66%A~90%A； 3.4~4.8 min， 90%A； 4.8~5 min， 90%A~66%A； 5~6 min， 66%A。柱温20 ℃，进样体积5 μL。

#### 1.2.4 质谱条件

质谱扫描模式采用电喷雾离子源，使用Scheduled-MRM模式。Scheduled-MRM是一种针对多组分分析中MRM自动智能分段的采集技术。系统根据提供的分析物列表、多反应监测参数条件和保留时间等，自动调整适宜的采集时间窗口监测每种分析物，实现不同离子对的动态扫描。将PEth同系物名称、离子化条件、保留时间等参数输入Scheduled-MRM方法列表，设定初始采集窗口为70 s并增加动态窗口扩展设置；使用目标周期时间模式，设置时间为2.5 s；喷雾气、辅助加热气、气帘气和碰撞气均为氮气，分别设置为241 kPa、552 kPa、159 kPa （即35 psi、80 psi、23 psi）和Medium，喷雾电压为-4500 V，离子源温度为600 ℃。

### 1.3 样本测定

用建立的LC-MS/MS法测定359名饮酒志愿者的PEth含量，以评价全血PEth与饮酒量的关系，同时分别采用两种不饮酒者全血样本作为空白对照。采用SPSS 25数据处理软件。采用Spearman相关性检验分析PEth之间以及与其他生化指标的相关性。定义*P*<0.05为具有统计学显著性意义。

## 2 结果与讨论

### 2.1 萃取体系的选择

根据现有的文献方法^[17⇓⇓-20]^，结合目标化合物的性质，考察了不同溶剂萃取体系（纯乙腈、乙腈-异丙醇、异丙醇、甲醇-甲基叔丁基醚-水等）的提取效果。与乙腈相比，异丙醇和甲醇-甲基叔丁基醚-水体系均表现出更好的萃取效率。后两者提取效率相当。与异丙醇单相萃取体系相比，甲醇-甲基叔丁基醚-水体系由于使用两相液液分层萃取原理，可以有效降低基质杂质的干扰，减小基质效应。同时甲基叔丁基醚沸点和黏度远小于异丙醇，便于后续吹干复溶等样本处理操作。

### 2.2 色谱条件的优化

#### 2.2.1 流动相

为保持一定的离子强度，改善峰形，减少拖尾，向流动相中加入一定浓度的乙酸铵，分别考察了0、2.5、5、7.5、10 mmol/L 5个乙酸铵浓度水平对保留时间及峰形的影响。以PEth18∶0/18∶1、PEth16∶0/20∶4、PEth18∶1/18∶1为例，对比不同乙酸铵浓度条件下的每种PEth的信号响应变化情况，结果如图1所示，当乙酸铵浓度为2.5 mmol/L时，所获得质谱信号响应最佳，故选择流动相中乙酸铵浓度为2.5 mmol/L。

**图1:**
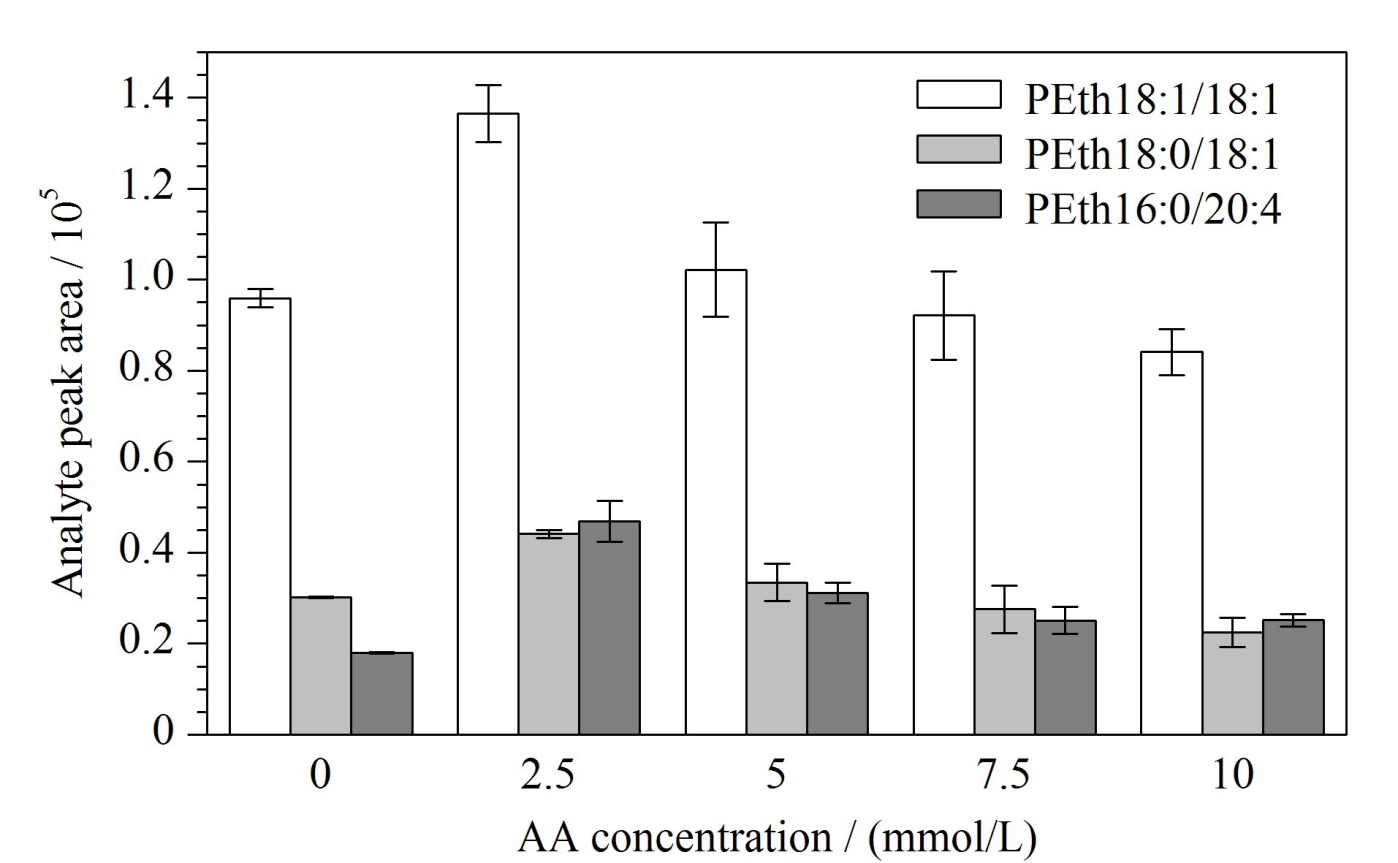
流动相中乙酸铵浓度对PEth响应的影响（*n*=3）

#### 2.2.2 色谱柱的选择

选取3根色谱柱进行对比，分别为Thermo HyPURITY C4 （150 mm×3 mm， 5 μm）， Waters XBridge C8 （100 mm×2.1 mm， 3.5 μm）和Waters XBridge C18 （100 mm×2.1 mm， 3.5 μm）色谱柱。C4和C8色谱柱虽有较短的保留时间，但是各PEth同系物分离度不足。C18色谱柱与待分析物具有更充分的相互作用，使峰形更尖锐，检测信号响应更好，同时采用的流动相条件能够充分保证待分析物洗脱出峰，保留时间相比前两种色谱柱虽略有延长，但在可接受范围内。综合考虑色谱峰形、保留时间、分离度、信号响应等因素，选择了C18柱。

### 2.3 质谱条件的优化

#### 2.3.1 仪器条件优化

在ESI负离子模式下采集PEth和内标的一级及二级扫描质谱图，图2为以PEth16∶0/18∶1为例的二级质谱扫描图及碎片离子断裂规律示意图。依据一、二级质谱信息可推断确定PEth监测母离子为[M-H]^-^离子，碎片离子（*m/z*见表1）为脂肪酸链残基和磷脂酰基团。由于现有标准品均为含有16∶0、18∶0、18∶1侧链的PEth，因此本方法主要监测含有相应侧链的PEth。每种PEth均至少选取两组离子对，优先考虑使用丰度最大的优势子离子作为定量离子，选取PEth的*sn*-3位特征子离子*m/z* 125作为定性通道。优化各PEth定量离子通道的母离子和子离子、对应内标、去簇电压、入口电压、碰撞能和出口电压等参数，优化后的质谱条件见表1。

**图2:**
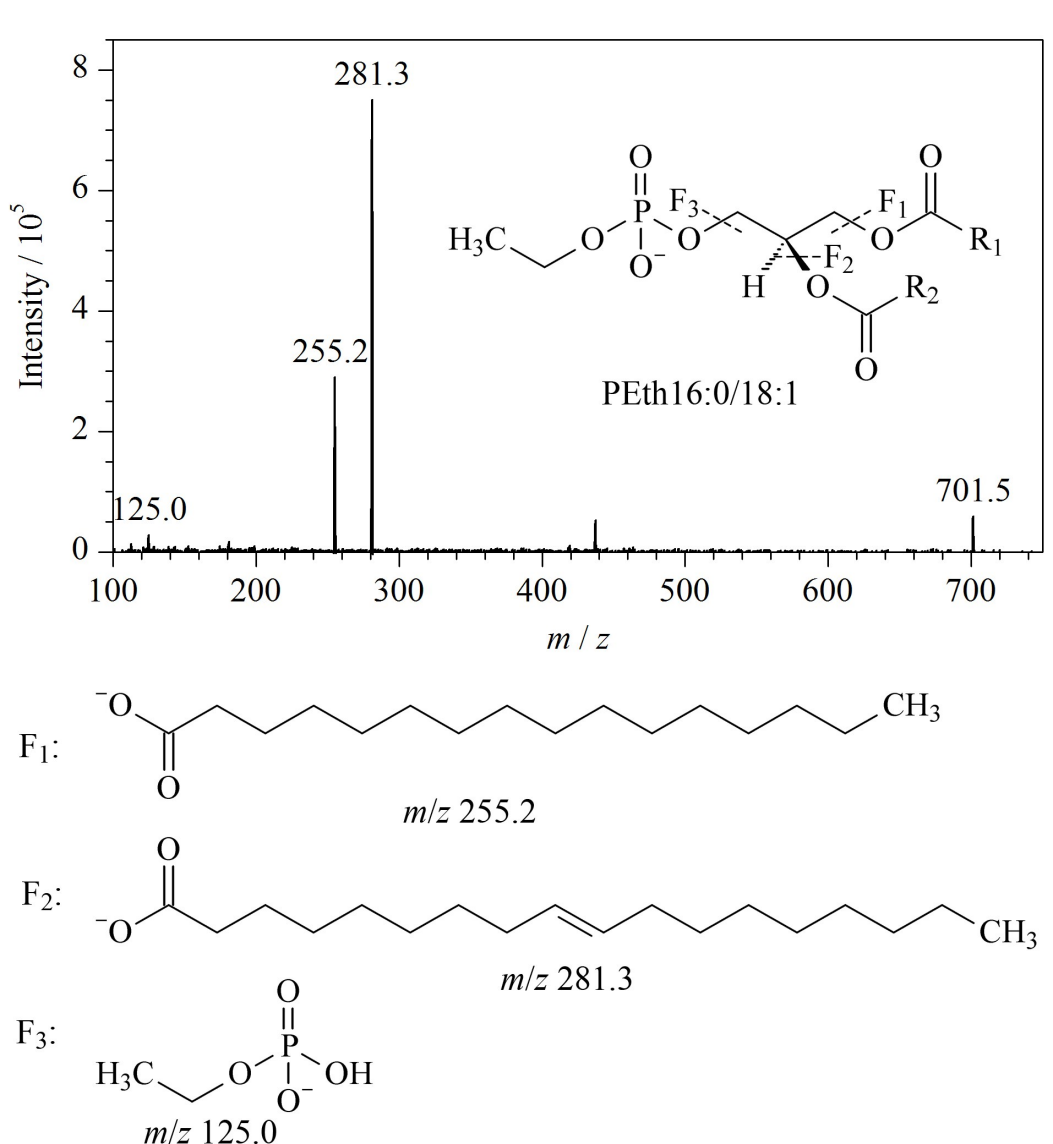
PEth16∶0/18∶1的二级质谱扫描图及质谱裂解途径

**表1:** PEth及内标物的串联质谱参数

PEth and IS	Precursor ion（*m/z*）	Product ion（*m/z*）	DP/V	EP/V	CE/eV	CXP/V
18∶1/18∶1	727.5	281.1	-5	-5	-45	-10
18∶1/18∶1	727.5	125.0	-5	-5	-45	-10
18∶0/18∶1	729.5	283.2	-5	-6	-45	-10
18∶0/18∶1	729.5	125.0	-5	-6	-45	-10
18∶0/18∶2	727.7	283.2	-5	-6	-42	-10
18∶0/18∶2	727.7	125.0	-5	-6	-45	-10
18∶0/20∶3	753.5	283.2	-5	-6	-45	-10
18∶0/20∶3	753.5	125.0	-5	-6	-45	-10
18∶0/20∶4	751.5	283.2	-5	-6	-45	-10
18∶0/20∶4	751.5	125.0	-5	-6	-45	-10
18∶0/20∶5	749.5	283.2	-5	-6	-45	-10
18∶0/20∶5	749.5	125.0	-5	-6	-45	-10
PEth and IS	Precursor ion（*m/z*）	Product ion（*m/z*）	DP/V	EP/V	CE/eV	CXP/V
18∶0/22∶4	779.6	283.2	-5	-6	-45	-10
18∶0/22∶4	779.6	125.0	-5	-6	-45	-10
18∶0/22∶5	777.5	283.2	-5	-6	-45	-10
18∶0/22∶5	777.5	125.0	-5	-6	-45	-10
18∶0/22∶6	775.5	283.2	-5	-6	-45	-10
18∶0/22∶6	775.5	125.0	-5	-6	-45	-10
18∶1/18∶2	725.7	279.2	-5	-6	-45	-10
18∶1/18∶2	725.7	125.0	-5	-6	-45	-10
18∶1/20∶1	755.6	281.2	-5	-6	-45	-10
18∶1/20∶1	755.6	125.0	-5	-6	-45	-10
18∶1/20∶2	753.5	281.2	-5	-6	-45	-10
18∶1/20∶2	753.5	125.0	-5	-6	-45	-10
18∶1/20∶3	751.5	281.2	-5	-6	-45	-10
18∶1/20∶3	751.5	125.0	-5	-6	-45	-10
18∶1/20∶4	749.5	281.2	-5	-6	-45	-10
18∶1/20∶4	749.5	125.0	-5	-6	-45	-10
18∶1/22∶4	777.5	281.2	-5	-6	-45	-10
18∶1/22∶4	777.5	125.0	-5	-6	-45	-10
18∶1/22∶5	775.5	281.2	-5	-6	-45	-10
18∶1/22∶5	775.5	125.0	-5	-6	-45	-10
18∶1/22∶6	773.5	281.2	-5	-6	-45	-10
18∶1/22∶6	773.5	125.0	-5	-6	-45	-10
16∶0/16∶0	675.5	255.2	-5	-9	-42	-18
16∶0/16∶0	675.5	125.0	-5	-9	-45	-9
16∶0/18∶1	701.5	281.3	-5	-6	-45	-15
16∶0/18∶1	701.5	125.0	-5	-6	-45	-15
16∶0/18∶2	699.5	279.2	-5	-6	-43	-16
16∶0/18∶2	699.5	125.0	-5	-6	-46	-9
16∶0/16∶1	673.5	255.2	-5	-6	-45	-10
16∶0/16∶1	673.5	125.0	-5	-6	-45	-10
16∶0/18∶0	703.5	283.2	-5	-6	-45	-10
16∶0/18∶0	703.5	125.0	-5	-6	-45	-10
16∶0/18∶3	697.5	255.2	-5	-6	-45	-10
16∶0/18∶3	697.5	125.0	-5	-6	-45	-10
16∶0/20∶2	727.7	255.2	-5	-6	-45	-10
16∶0/20∶2	727.7	125.0	-5	-6	-45	-10
16∶0/20∶3	725.7	255.2	-5	-6	-45	-10
16∶0/20∶3	725.7	125.0	-5	-6	-45	-10
16∶0/20∶4	723.5	255.2	-5	-9	-44	-9
16∶0/20∶4	723.5	125.0	-5	-6	-45	-10
16∶0/20∶5	721.5	255.2	-5	-6	-45	-10
16∶0/20∶5	721.5	125.0	-5	-6	-45	-10
16∶0/22∶4	751.5	255.2	-5	-6	-45	-10
16∶0/22∶4	751.5	125.0	-5	-6	-45	-10
16∶0/22∶5	749.5	255.2	-5	-6	-45	-10
16∶0/22∶5	749.5	125.0	-5	-6	-45	-10
16∶0/22∶6	747.5	255.2	-5	-6	-45	-10
16∶0/22∶6	747.5	125.0	-5	-6	-45	-10
16∶1/18∶0	701.5	283.2	-5	-6	-45	-10
16∶1/18∶0	701.5	125.0	-5	-6	-45	-10
16∶1/18∶1	699.5	281.2	-5	-6	-45	-10
16∶1/18∶1	699.5	125.0	-5	-6	-45	-10
14∶0/16∶0	647.5	255.2	-5	-6	-45	-10
PEth and IS	Precursor ion（*m/z*）	Product ion（*m/z*）	DP/V	EP/V	CE/eV	CXP/V
14∶0/16∶0	647.5	125.0	-5	-6	-45	-10
14∶0/18∶1	673.5	281.2	-5	-6	-45	-10
14∶0/18∶1	673.5	125.0	-5	-6	-45	-10
d5-16∶0/18∶1	706.5	281.1	-5	-10	-44	-15
d5-16∶0/18∶1	706.5	130.0	-5	-10	-49	-15
d5-16∶0/18∶2	704.5	279.3	-5	-10	-46	-15
d5-16∶0/18∶2	704.5	130.0	-5	-10	-41	-15
d5-18∶0/18∶2	732.5	283.2	-5	-10	-43	-15
d5-18∶0/18∶2	732.5	130.0	-5	-10	-45	-15
d5-18∶0/18∶1	734.5	283.3	-5	-10	-45	-15
d5-18∶0/18∶1	734.5	130.0	-5	-10	-48	-15
d5-16∶0/20∶4	728.3	303.2	-5	-10	-39	-15
d5-16∶0/20∶4	728.3	130.2	-5	-10	-44	-15
Phosphatidylbutanol 18∶1/18∶1（Pbut 18∶1/18∶1）	755.6	281.1	-5	-10	-47	-20
Phosphatidylbutanol 18∶1/18∶1（Pbut 18∶1/18∶1）	755.6	153.0	-5	-10	-50	-20
Phosphatidylpropanol 16∶0/16∶0（Pprop 16∶0/16∶0）	689.6	255.2	-5	-6	-41	-20
Phosphatidylpropanol 16∶0/16∶0（Pprop 16∶0/16∶0）	689.6	139.0	-5	-6	-45	-20

DP：declustering potential；EP：entrance potential；CE：collision energy；CXP：cell exit potential.

#### 2.3.2 采集模式的选择

常规MRM中使用过多的并行离子通道会严重挤占驻留时间、降低分析重现性，Scheduled-MRM则改变了以往普通MRM在任何时间点都持续开启监测的传统做法。Scheduled-MRM对质谱信号采集周期进行合理分段，为不同的离子通道选择专属开启时间，实现不同离子对的动态扫描。该技术能够有效减少单位时间内离子对监测并行数量，克服离子通道过多造成驻留时间不足的问题，避免非保留时间的冗余监测，从而增加每个目标物离子通道的有效驻留时间，显著提升检测灵敏度和数据采集质量。Scheduled-MRM模式下PEth标准品及内标的色谱图如图3所示。

**图3:**
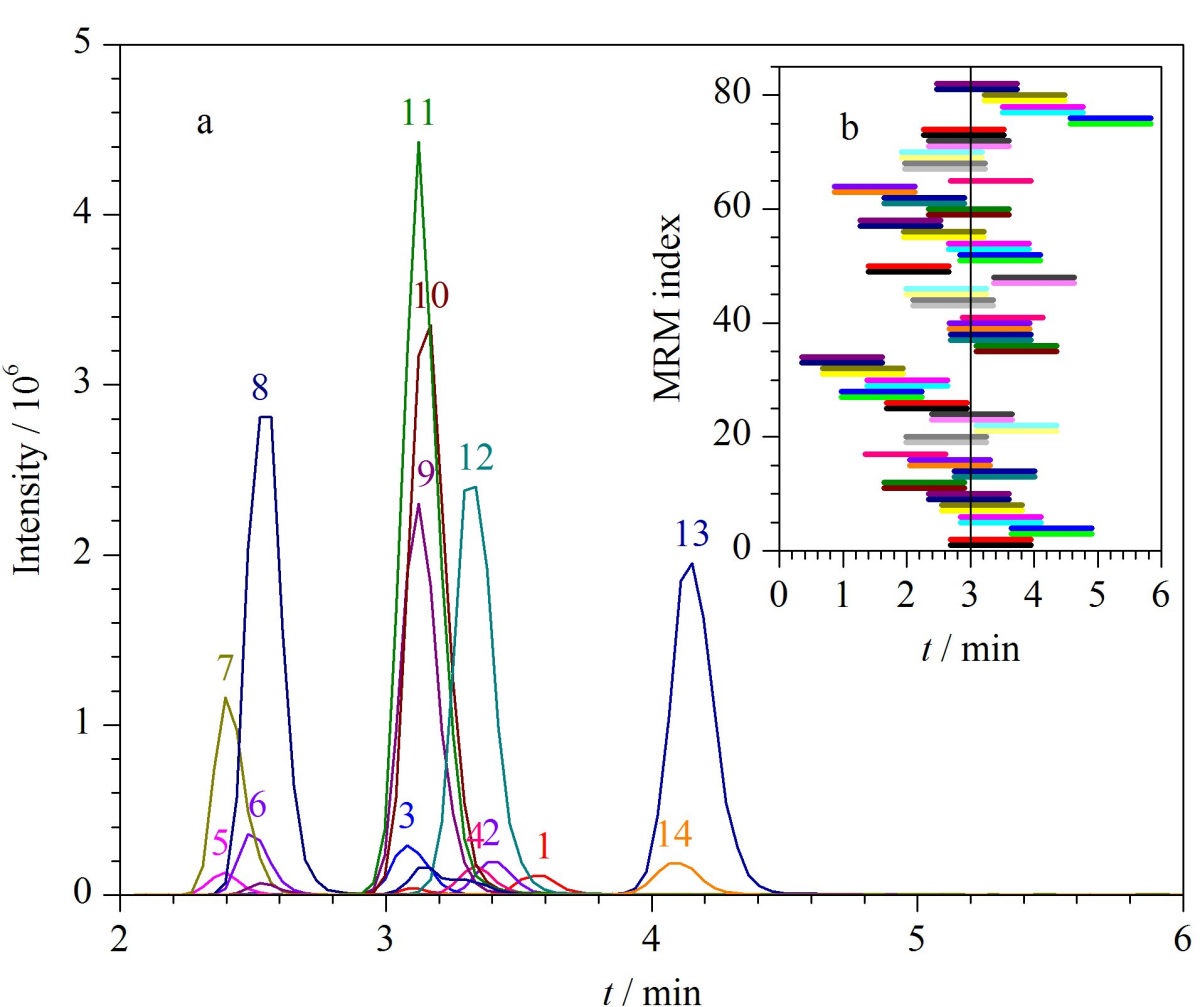
智能化分时区间-负离子多反应监测PEth标准品及内标的色谱图

### 2.4 方法学验证

#### 2.4.1 PEth的特异性

饮酒者全血样本及禁酒者全血样本的LC-MS/MS分析结果如图4所示。饮酒者样本中PEth保留时间为2.0~5.0 min，出峰时间内没有明显杂质峰出现，基线噪声低，无内源性物质干扰，而禁酒者全血样本在相应位置无峰出现，表明禁酒者体内无PEth检出。需要指出的是磷脂酸与PEth虽互为同分异构体，但仅PEth能够实现表1中定量和定性离子通道的同时出峰。此外，如图4b所示，空白血样本在检测时间窗口内无*sn*链相关峰检出，表明磷脂酸的*sn*链子离子在所建方法检测时间窗口内不会对PEth分析产生干扰，方法特异性较好。

**图4:**
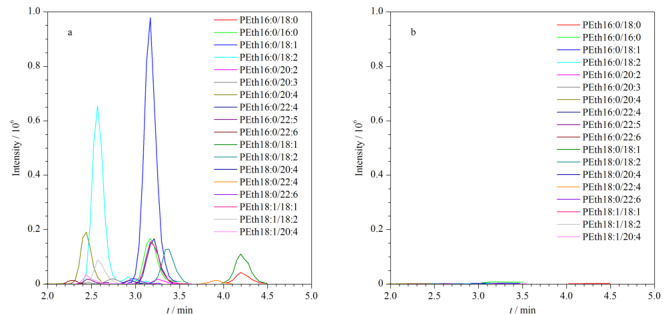
MRM模式下实际血样的LC-MS/MS谱图

#### 2.4.2 线性关系、检出限、定量限、准确度和精密度

重复测定5次PEth系列混合标准溶液，以PEth的质量浓度为X轴，待分析物与内标峰面积的比值为Y轴，进行线性回归分析。稀释混合标准溶液，评估检出限（LOD）和定量限（LOQ）（分别定义为信噪比为3和10的浓度）。结果显示，5次实验的线性相关系数（*R*^2^）大于0.9999，表面线性关系良好，线性范围为10~2500 ng/mL， LOD为0.7~2.8 ng/mL，LOQ为2.2~9.4 ng/mL（见表2）。

**表2:** 目标化合物的线性方程、相关系数、线性范围、检出限、定量限

PEth	Linear equation	*R*^2^	Linear range/（ng/mL）	LOD/（ng/mL）	LOQ/（ng/mL）
16∶0/16∶0	*Y*=0.0056*X*+0.0391	0.9998	10-2500	2.6	8.8
16∶0/18∶1	*Y*=0.0038*X*+0.0245	0.9999	10-2500	1.5	5.1
16∶0/18∶2	*Y*=0.0042*X*+0.0463	0.9999	10-2500	1.3	4.3
16∶0/20∶4	*Y*=0.0038*X*+0.0289	0.9998	10-2500	2.8	9.4
18∶1/18∶1	*Y*=0.0209*X*+0.1607	0.9998	10-2500	1.4	4.7
18∶0/18∶1	*Y*=0.0050*X*+0.0494	0.9998	10-2500	1.3	4.5
18∶0/18∶2	*Y*=0.0052*X*+0.0757	0.9999	10-2500	0.7	2.2

*Y*：peak area ratio of analyte to internal standard；*X*：mass concentration of PEth，ng/mL.

向两种不饮酒者空白全血样本（blood 1和blood 2）中分别加入100、250、500 ng/mL的标准溶液，每种加标样本制备3个平行管，测定加入标准溶液前后的全血样本，计算目标物的回收率及相对标准偏差（RSD），共重复测定5个批次。结果表明加标回收率在91.0%~102.2%之间，日内精密度和日间精密度的RSD值为0.4%~7.4%（见表3）。

**表3:** 目标化合物的加标回收率及精密度

PEth	Blood sample	Added/ （ng/mL）	Found/ （ng/mL）	Recovery/% （*n*=5）	Intra-day RSD/% （*n*=3）	Inter-day RSD/% （*n*=5）
16∶0/16∶0	1	100	96.5	96.5	2.1	5.7
		250	227.4	91.0	3.5	2.8
		500	497.4	99.5	1.1	5.3
	2	100	96.2	96.2	1.6	4.5
		250	234.0	93.6	2.7	4.0
		500	483.7	96.7	2.1	2.8
16∶0/18∶1	1	100	100.3	100.3	2.4	2.6
		250	251.6	100.6	2.3	2.0
		500	508.9	101.8	0.4	1.1
	2	100	100.7	100.7	2.0	2.1
		250	254.5	101.8	2.8	1.8
		500	506.7	101.3	4.5	1.9
16∶0/18∶2	1	100	100.4	100.4	1.2	3.1
		250	250.2	100.1	1.9	2.7
		500	511.0	102.2	5.5	1.4
	2	100	99.1	99.1	3.7	4.8
		250	233.3	93.3	2.4	4.8
		500	486.4	97.3	3.4	4.9
16∶0/20∶4	1	100	96.5	96.5	1.0	4.6
		250	247.1	98.9	3.8	4.3
		500	488.9	97.8	1.2	4.4
	2	100	96.6	96.6	3.0	5.8
		250	250.6	100.2	2.8	4.0
		500	476.1	95.2	1.3	4.4
18∶1/18∶1	1	100	101.2	101.2	4.9	3.3
		250	251.1	100.4	0.4	2.9
		500	500.3	100.1	1.0	5.4
	2	100	101.2	101.2	2.4	4.3
		250	251.7	100.7	2.6	5.9
		500	509	101.8	2.9	3.2
18∶0/18∶1	1	100	100.7	100.7	3.4	3.1
		250	235.6	94.2	3.6	4.3
		500	505.1	101.0	2.7	6.5
	2	100	100.3	100.3	7.4	5.9
		250	235.6	94.2	3.5	3.3
		500	489.5	97.9	0.9	2.7
18∶0/18∶2	1	100	100.8	100.8	0.4	4.5
		250	252.5	101.0	2.5	6.3
		500	493.6	98.7	3.3	3.6
	2	100	102.0	102.0	3.2	7.0
		250	243.6	97.4	2.6	8.3
		500	509.5	101.9	2.6	3.8

#### 2.4.3 基质效应

本研究还对方法的基质效应进行了考察。分别对经过相同前处理步骤的标准溶液和人血样本中稳定同位素标记内标物的绝对响应进行了比较，未见明显离子抑制或增强。此外，我们采用柱后流动灌注法进一步对潜在基质效应进行了监测，以注射针泵持续注射250 ng/mL标准溶液，色谱进样吸取空白人全血基质样本，观察质谱采集信号变化，结果显示在一针分析运行周期内未观察到质谱信号的明显变化，进一步证明所建方法没有明显基质效应。

### 2.5 方法的人群应用

将所建方法应用于实际人群样本酒精摄入状况评估。根据PEth的甘油骨架结构特点以及两条脂肪酸侧链上碳原子数和不饱和双键数量，同时依据已有的部分PEth标准品，推算了PEth同系物的质谱离子通道和色谱保留时间，并饮酒人群样本中检出18种PEth （PEth16∶0/16∶0、PEth16∶0/18∶1、PEth16∶0/18∶2、PEth16∶0/20∶4、PEth18∶1/18∶1、PEth18∶0/18∶1、PEth18∶0/18∶2、PEth18∶0/20∶4、PEth18∶0/22∶4、PEth18∶0/22∶6、PEth18∶1/18∶2、PEth18∶1/20∶4、PEth16∶0/18∶0、PEth16∶0/20∶2、PEth16∶0/20∶3、PEth16∶0/22∶4、PEth16∶0/22∶5、PEth16∶0/22∶6）。其中7种PEth具有市售标准品（PEth16∶0/16∶0、PEth16∶0/18∶1、PEth16∶0/18∶2、PEth16∶0/20∶4、PEth18∶1/18∶1、PEth18∶0/18∶1、PEth18∶0/18∶2），可直接通过各自的标准曲线进行定量，其余11种PEth因无市售标准品故使用与其保留时间最接近并且具有标准品的PEth同系物标准曲线进行定量。在内标物质选择上，我们优先使用稳定同位素标记的PEth作为对应内标，对于无市售同位素内标物的PEth则依据结构类似、色谱峰保留时间相近原则，选择相应磷脂酰丙醇或磷脂酰丁醇等结构近似物质作为对应内标（见表1）。

本研究共收集359名在日常生活中有规律饮酒习惯的志愿者（男性350名，女性9名），年龄在19~90岁之间，用所建方法测定所有志愿者的全血PEth水平。结果显示，18种PEth均在相应工作曲线线性范围内，样品中的总PEth质量浓度范围为51.13 ng/mL~2.89 μg/mL，平均为363.16 ng/mL。如图5所示，研究发现志愿者体内PEth16∶0/18∶1、16∶0/18∶2是含量占优势地位的两种主要PEth同系物，平均质量浓度分别为74.21和48.75 ng/mL，各约占总PEth的20.43%和13.42%，同时PEth16∶0/18∶1、16∶0/18∶2、18∶0/18∶2、18∶0/18∶1、18∶0/20∶4、16∶0/20∶4、16∶0/16∶0这6种同系物含量占PEth总含量的76%以上，能有效反映样本的总PEth含量。

**图5:**
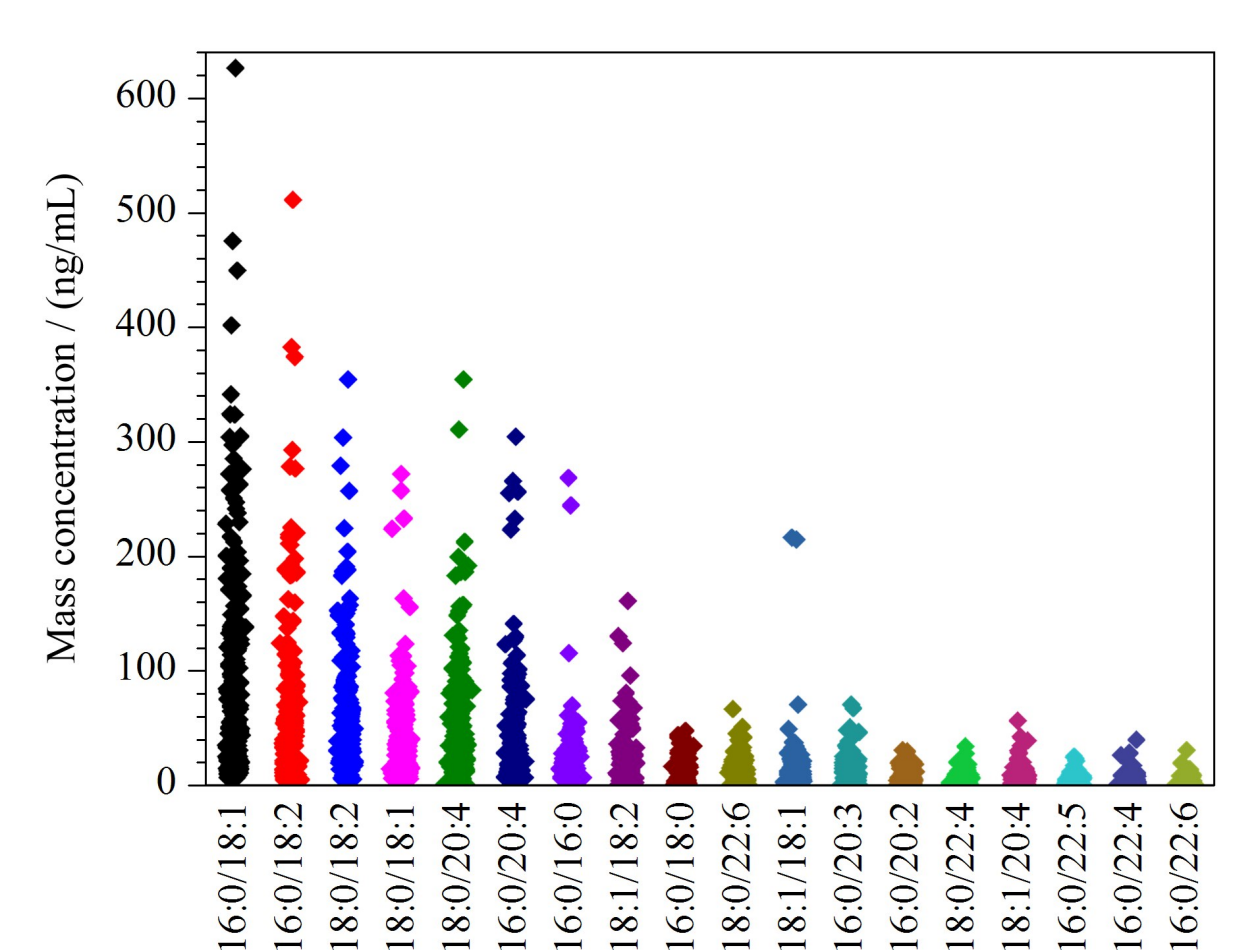
饮酒状况研究志愿者中18种PEth水平的分布（*n*=359）

我们用所建方法测定了有规律饮酒习惯的志愿者人群，并以PEth含量量化反映日常酒精摄入状态，在此基础上使用Spearman相关性分析探索酒精摄入与临床肝肾功能相关生化指标的关系。结果如表4所示，PEth中占比最大的两种同系物即PEth16∶0/18∶1和16∶0/18∶2之间相关性良好，同时与临床现有酒精摄入生物学标志物*γ*-谷氨酰转肽酶（*γ*-GGT）呈显著正相关而与人群年龄、性别、身体质量指数等体征水平无关，表明PEth是酒精摄入的特异性代谢物。总PEth与临床常用肝肾功能指标（*γ*-GGT、谷草转氨酶（AST）、内生肌酐清除率（Ccr）、白蛋白（ALB）等）、肝癌标志物（甲胎蛋白）等均呈统计学显著正相关关系。人血清中*γ*-GGT和AST等主要来自肝脏，是反应肝功能损伤的重要指标，有文献报道重度酗酒者血清中*γ*-GGT明显升高^[21]^，而甲胎蛋白与肝癌及多种肿瘤的发生发展密切相关，临床上作为原发性肝癌的血清标志物^[22,23]^。上述结果说明乙醇摄入在一定程度上损伤肝功能，可能会增加肝癌患病风险。同时也表明PEth作为能够量化反映酒精摄入的客观指标，在临床疾病的预防和诊治过程中具有潜在的应用价值。

**表4:** PEth水平与传统临床指标的非参数Spearman相关系数

Item	PEth16∶0/18∶1	PEth16∶0/18∶2	SUM-PEth
Age	-0.062	-0.072	-0.047
Gender	-0.029	-0.026	-0.031
BMI	0.034	0.002	0.024
PEth16∶0/18∶1	-	0.944^**^	0.983^**^
PEth16∶0/18∶2	0.944^**^	-	0.959^**^
Crea	-0.150^**^	-0.154^**^	-0.149^**^
Ccr	0.134^*^	0.123^*^	0.135^*^
*γ*-GGT	0.353^**^	0.233^**^	0.331^**^
AST	0.167^**^	0.121^*^	0.167^**^
ALB	0.124^*^	0.113^*^	0.127^*^
AFP	0.236^**^	0.233^**^	0.246^**^

BMI：body mass index；Crea：creatinine；Ccr：endogenous creatinine clearance；*γ*-GGT：*γ*-glutamyltransferase；AST：aspartate transaminase；ALB：albumin；AFP：α-fetoprotein. ** *P*<0.001；* *P*<0.005.

## 3 结论

本研究建立了一种基于智能化分时区间-多反应监测采集技术的PEth液相色谱-串联质谱分析方法，一次进样可同时实现对18种PEth同系物的准确测定。该方法样品前处理步骤简单，具有较高的灵敏度、较好的准确性和精密度。所建方法可用于人群实际样本的测定，能够准确反映PEth同系物分布和酒精摄入状态，有望为临床酒精摄入监测及干预效果评估提供新的、更为客观的技术手段。
